# Supplementary material for: Mechanistic models of asymmetric hand-over-hand translocation and nucleosome navigation by CMG helicase
Source: Nat Commun. 2025 Nov 21;16:10304. doi: 10.1038/s41467-025-65232-x (PMC12639105; doi:10.1038/s41467-025-65232-x)
Supplement: Supplementary file 18 — Source data [file 41467_2025_65232_MOESM18_ESM.zip › SourceData/SourceData/Fig5C/displacement/max_tracking_resid009.pdf]

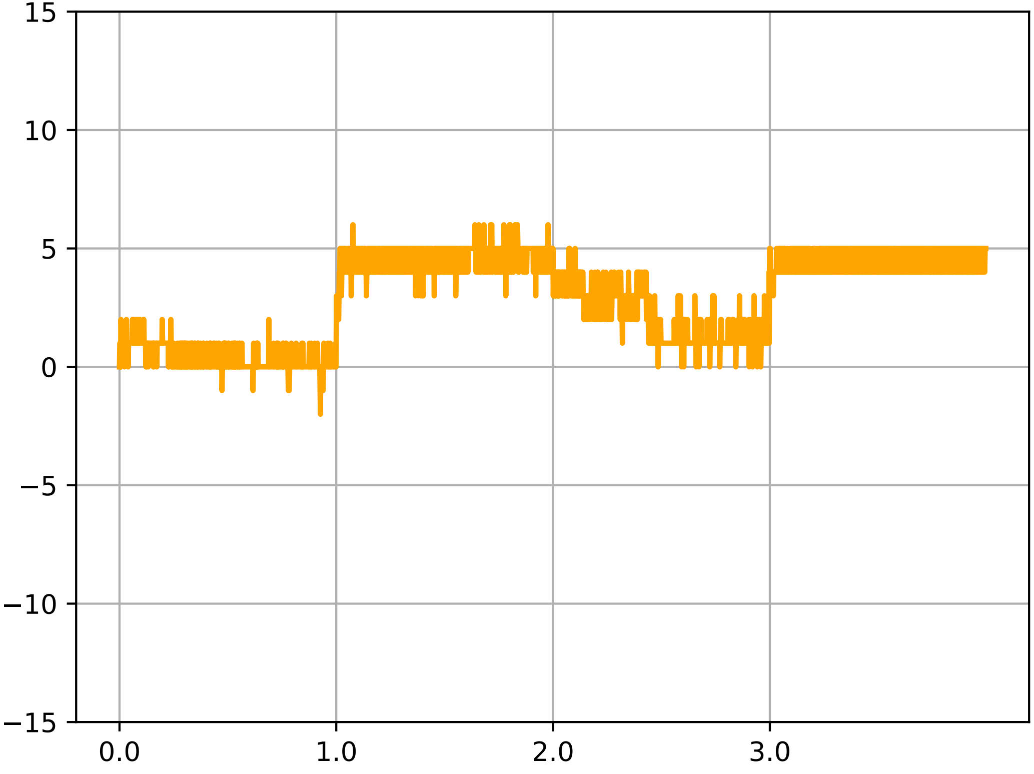

15
10
5
0
−5
−10
−15
0.0
1.0
2.0
3.0
